# How do working-memory-related demand, reasoning ability and aversive reinforcement modulate conflict monitoring?

**DOI:** 10.3389/fnhum.2014.00210

**Published:** 2014-04-11

**Authors:** Anja Leue, Bernd Weber, André Beauducel

**Affiliations:** ^1^Institute of Psychology, University of BonnBonn, Germany; ^2^Department of Epileptology, University Hospital BonnBonn, Germany; ^3^Center for Economics and Neuroscience, University of BonnBonn, Germany; ^4^Life and Brain Center, Department of NeuroCognition, University of BonnBonn, Germany

**Keywords:** working memory, cognitive demand, conflict monitoring, reasoning ability, aversive reinforcement

## Abstract

Conflict monitoring is a process of stimulus evaluation and a pre-requisite for subsequent recruitment of cognitive control and behavioral adaptations. This study investigated how experimentally manipulated working-memory-related cognitive demand and aversive reinforcement modulate individual differences of conflict monitoring intensity and behavioral adjustments. Individual differences were assessed by means of an anxiety-related trait dimension (trait-BIS) and by means of reasoning abilities—a core determinant of intelligence. Moreover, we investigated the special role of verbal reasoning ability and figural reasoning ability for the modulation of the conflict monitoring intensity. Ninety participants performed a go/nogo task with four conditions each comprising a combination of low vs. high working-memory-related cognitive demand and low vs. high aversive reinforcement. No effect of aversive reinforcement was observed for the N2 amplitude. The fronto-central nogo N2 amplitude was more pronounced for high demand vs. low demand suggesting that cognitive demand served as an aversive costly event. Higher total reasoning abilities were associated with more intense conflict monitoring and shorter response times with increasing aversive reinforcement (defined as verbal error-feedback vs. monetary loss). Individuals with higher trait-BIS scores demonstrated a more intense conflict monitoring even in conditions with low aversive reinforcement and also a more cautious responding (i.e., response times slowing) with increasing aversive reinforcement indicating a focus on negative feedback prevention. The findings provide evidence for the conflict monitoring theory and suggest that working-memory-related demand overrules the impact of aversive reinforcement on conflict monitoring intensity. Reasoning abilities and anxiety-related traits go along with an intensification of conflict monitoring but differences in the flexibility of behavioral adjustment.

## Introduction

The Anterior Cingulate Cortex (ACC) is an important neural structure in the medial frontal cortex and plays a central role for the integration of negative affect, pain, and cognitive control (e.g., Bekker et al., 2005; Shackman et al., 2011). Models on conflict monitoring and error monitoring have been developed to derive predictions on adjustments in ACC-related cognitive control (Botvinick et al., 2004; Yeung and Cohen, 2006; Botvinick, 2007; Shenhav et al., 2013) and error detection (Ullsperger and von Cramon, 2003; Yeung et al., 2004; Klein et al., 2007; Moser et al., 2013). Based on these models, the involvement of the dorsal ACC or the dorsal midcingulate cortex (MCC) in neural responses to working-memory-related cognitive demand, cognitive control (e.g., Vogt, 2009; Shenhav et al., 2013; Hernandez Lallement et al., 2014), and negative feedback or aversive reinforcement (Amodio et al., 2008; Leue et al., 2009, 2012b; Riesel et al., 2012) has been intensively investigated. Studying the role of determinants that modulate the intensity of conflict monitoring and error monitoring facilitates our knowledge on how individuals recruit and adjust cognitive control (Braver et al., 2007; Braver, 2012; Weldon et al., 2013) and compensate inefficiency i.e., for information processing (e.g., Moser et al., 2013). However, effects of working-memory-related cognitive demand and aversive reinforcement on conflict monitoring have not yet been investigated simultaneously. Thus, the present study aimed at investigating these combined effects on conflict monitoring because it is likely that working-memory-related demand and aversive reinforcement occur simultaneously when conflict monitoring is required. Since effects of working-memory-related demand and aversive reinforcement are likely to be modulated by individual differences in reasoning ability and anxiety-related differences, the present study aimed at investigating these effects of cognitive and non-cognitive individual differences on conflict monitoring.

The ACC—and in particular the dorsal ACC—has been shown to monitor task difficulty (Botvinick et al., 2004; Ridderinkhof et al., 2004; Botvinick and Rosen, 2009) resulting in greater ACC activity in high cognitive demand conditions than in low cognitive demand conditions (Botvinick et al., 2009). Accordingly, higher task difficulty and required cognitive demand should intensify conflict monitoring but individuals might differ in the efficiency with which they deal with situations that require higher cognitive demand (see below) and in their strategies to discount effort (Botvinick et al., 2009; Vogt, 2009; Shenhav et al., 2013).

Task difficulty during conflict monitoring could come along with variations of the go-nogo ratio in go/nogo tasks (Schacht et al., 2009; Leue et al., 2012a). In case of an asymmetric go-nogo ratio (e.g., 80% go vs. 20% nogo stimuli) go responses are predominant and, thus, making adjustments in the motor plan more difficult. Moreover, the manipulation of cognitive demand by means of the go-nogo ratio probably parallels to oddball paradigms that evoke more stimulus-related aspects of conflict monitoring and subsequent cognitive control (Folstein and Van Petten, 2008) along with different requirements to adjust the motor plan of responses (Hewig et al., 2011). Although de-confounding conditions of cognitive demand and aversive reinforcement, Leue et al. (2012a) found that the nogo N2 amplitude was more pronounced in high vs. low cognitive demand conditions at posterior sites. This finding suggests that the go-nogo ratio activates the stimulus-driven attentional system in posterior areas of the brain and bottom-up control (Corbetta and Shulman, 2002; Eysenck et al., 2007). Leue et al. (2012a) could not rule out that manipulating cognitive demand by means of the go-nogo ratio might have been a rather weak manipulation of cognitive demand and, thus, might have facilitated evidence for a predominant role of aversive reinforcement during conflict monitoring especially in individuals with higher anxiety scores as predicted in the revised reinforcement-sensitivity-theory (Corr, 2004, 2008). Thus, it was important to identify an independent variable that allows for a more intense manipulation of cognitive demand. Recent models on conflict monitoring proposed working memory load as a promising measure of cognitive demand during conflict monitoring and cognitive control (Gray and Braver, 2002; Botvinick, 2007; Braver, 2012). Therefore, we used working memory load as a manipulation of cognitive demand in the present study in order to investigate the interplay of cognitive demand and aversive reinforcement on conflict monitoring.

Functional Magnetic Resonance Imaging (fMRI) studies indicate that the dorsal ACC is prominently involved in the processing of cognitively demanding events that require working memory (e.g., Gray and Braver, 2002; Braver et al., 2007; Hernandez Lallement et al., 2014). These fMRI studies suggest that conflict monitoring is more intense when working-memory-related cognitive demand is high. In accordance with relevant theoretical accounts (Yeung and Cohen, 2006; Botvinick, 2007; Eysenck et al., 2007), it can be expected that those conflict monitoring tasks that require substantial amounts of working memory load are likely to activate the ACC during conflict monitoring. However, until now no evidence for an intensified ACC-related conflict monitoring in a working-memory-related condition has been shown for the N2 component of the event-related-potential (ERP). The N2-component has been introduced as an indicator of conflict monitoring that occurs about 250 ms post-stimulus with a most negative peak at fronto-central sites (Donkers and van Boxtel, 2004; Yeung et al., 2004; Amodio et al., 2008; Schacht et al., 2010; Leue et al., 2012a). Several studies demonstrated dipole generators of the N2 component in the ACC (Nieuwenhuis et al., 2003; Amodio et al., 2008; Aarts and Pourtois, 2010; Leue et al., 2012a) also supporting the proposal that the ACC and more specifically the dorsal ACC is prominently involved in conflict monitoring and error monitoring, respectively. Moreover, the ACC-related N2 component is more pronounced following rarely occurring nogo trials compared to go trials in go/nogo tasks. Consequently, the N2 component will be used as an indicator of conflict monitoring in the present study and we expect that the ACC-related N2 component is sensitive to variations of working-memory-related cognitive demand during conflict monitoring. Since working-memory-related cognitive demand and aversive reinforcement have been mostly investigated in separate conflict monitoring studies, less is known about the combined effects of working-memory-related cognitive demand and aversive reinforcement on conflict monitoring.

**Figure 1:**
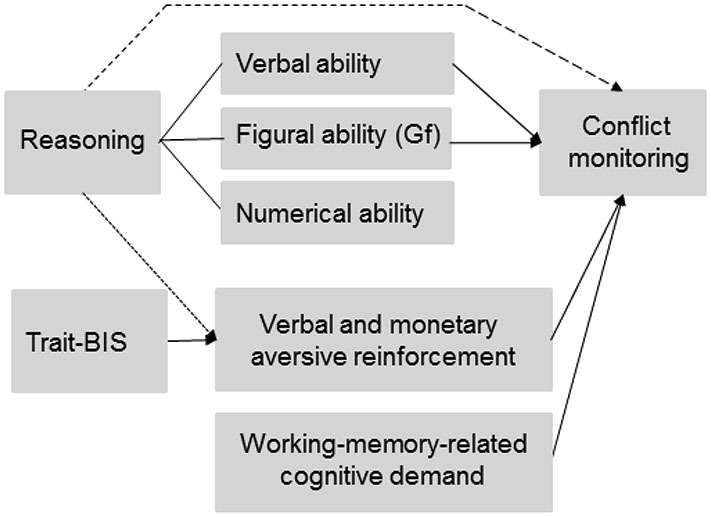
**Predicted and observed associations between individual differences (reasoning ability and trait-BIS), experimentally manipulated working-memory-related cognitive demand, and aversive reinforcement on conflict monitoring**. The dotted line represents a link between reasoning ability and experimentally varied aversive reinforcement on conflict monitoring that was not initially predicted but observed in the data. The dashed line between reasoning and conflict monitoring was predicted but not observed in the data. Gf = General fluid intelligence.

The manipulation of conflict monitoring intensity by means of working memory load implies that those individual differences that are associated with working memory capacity (WMC) like reasoning ability and general fluid intelligence (Gf; Kyllonen and Christall, 1990; Süß et al., 2002; Burgess and Braver, 2010) might affect conflict monitoring intensity. Reasoning ability (i.e., a core determinant of intelligence that is related to verbal, numerical, and figural abilities) as well as Gf (i.e., the ability to solve problems, to recognize patterns and to learn, Cattell, 1971) are closely related to individual differences of working-memory capacity (Kyllonen and Christall, 1990; Engle et al., 1999; Süß et al., 2002). It has been emphasized that reasoning ability closely matches Gf (Cattell, 1987; Süß et al., 2002) and it has been shown that verbal and numerical abilities (Süß et al., 2002) are also relevant for the investigation of WMC (Colom et al., 2004). Therefore, we will use the term “total reasoning” subsequently in order to separate different aspects of total reasoning like verbal, numerical, and figural reasoning (Süß et al., 2002). Thus, total reasoning can be decomposed according to the verbal, numerical or figural content (Figure 1), on which the reasoning tasks are based (Beauducel et al., 2001). Engle et al. (1999) argued that Gf and WMC reflect the ability to keep a representation active, especially when interference and distraction occur. Since conflict monitoring has been related to cognitive resources required to deal with the interference caused by distractors (Weldon et al., 2013), we investigated whether Gf is closely related to conflict monitoring as has been suggested in prior fMRI studies (Gray et al., 2005; Burgess and Braver, 2010). Moreover, individuals with higher WMC have been described to engage in a more flexible adjustment of cognitive control (Weldon et al., 2013). Since a positive association of WMC and reasoning has been demonstrated (Kyllonen and Christall, 1990; Oberauer et al., 2007) we predict that individuals with higher total reasoning ability should demonstrate more flexibility in tasks that require intense conflict monitoring and subsequent behavioral adjustments. A more flexible adjustment of behavior implies that individuals with higher reasoning ability should be better prepared for fast and correct responses. Accordingly, we expect that the enhanced adjustment flexibility of individuals with higher total reasoning ability is related to an intensified ACC-related conflict monitoring (i.e., more negative N2 amplitude) and better subsequent behavioral performance in tasks that require cognitive control. Since total reasoning closely matches Gf, we also expected individuals with higher Gf scores to show a more pronounced N2-related conflict monitoring intensity. This association of Gf and conflict monitoring should be observable especially for the figural reasoning tasks (e.g., matrices) that have been proposed as measures of Gf (Cattell, 1987). Moreover, Süß et al. (2002) found a strong association between verbal/numerical WMC and verbal reasoning. We therefore expect that verbal reasoning is related to conflict monitoring in addition to total reasoning and Gf especially when the task requirements are verbally coded. Similarly, figural reasoning or Gf should be related to conflict monitoring when the task is primarily based on figural material. These predictions on reasoning and conflict monitoring imply that working-memory-related individual differences serve in its own rights as a determinant of conflict monitoring intensity and, thus, independently of an experimental manipulation of working-memory-related cognitive demand (Figure 1).

Regarding the role of anxiety-related traits during conflict monitoring, we refer to the revised reinforcement-sensitivity-theory (rRST; Gray and McNaughton, 2000; Corr, 2008; Leue and Beauducel, 2008), which postulates individual differences with regard to conflict detection and resolution. In rRST, aversive reinforcement has been hypothesized to intensify conflict detection and resolution. Specifically, the behavioral inhibition system (BIS) as a neural system is thought to detect conflict information. Accordingly, individuals with higher anxiety-related scores (named as trait-BIS, Carver and White, 1994) have been predicted to show a more active BIS resulting in an intensified conflict monitoring especially when aversive reinforcement is likely to occur following erroneous responses (e.g., Corr, 2008). This prediction has been confirmed in prior N2 studies that applied aversive verbal reinforcement and monetary loss, respectively, as feedback in conflict monitoring tasks (e.g., Amodio et al., 2008; Leue et al., 2012a,b). Therefore, we expected in this study that higher vs. lower Trait-BIS individuals demonstrate a more intense conflict monitoring and that this intensification of conflict monitoring is most pronounced in higher vs. lower Trait-BIS individuals in conditions with more intense aversive reinforcement (i.e., monetary loss). Thus, contrary to individual differences of reasoning ability an intensification of conflict monitoring in higher trait-BIS individuals is related to situations with externally applied aversive reinforcement (Figure 1).

To summarize, the manipulation of cognitive demand by means of working memory demand was combined with an investigation of individual differences in total reasoning, verbal reasoning, and Gf because these abilities are expected to modulate the conflict monitoring intensity in situations that require working-memory-related cognitive demand. Moreover, the manipulation of aversive reinforcement was combined with an investigation of individual differences in the sensitivity for aversive reinforcement (trait-BIS), which is important, because trait-BIS is expected to modulate the aversive reinforcement effect. Thus, the experimental manipulations of aversive reinforcement and cognitive demand are paired with those individual differences that might modulate the respective treatment intensity at the individual level.

By using a go/nogo task that proved valid for the investigation of the N2-component as an index of the conflict monitoring intensity (Amodio et al., 2008; Leue et al., 2012a,b), we manipulated aversive reinforcement by means of negative feedback (low vs. high) and cognitive demand by means of the required working memory load (low vs. high). Presuming a fronto-central N2 topography, we hypothesized that (a) the N2 component should be more negative to nogo than to go stimuli because rarely occurring nogo stimuli require more intense stimulus evaluation and comparison than responding to frequently occurring go stimuli suggesting a more intense conflict monitoring to nogo stimuli compared to go stimuli (e.g., Donkers and van Boxtel, 2004; Amodio et al., 2008; Leue et al., 2012a). We expected the nogo N2 component to be (b) more negative for high-demand than low-demand because in the high working-memory-related demand conditions three different nogo-stimuli have to be kept in mind and compared to one go stimulus whereas a single nogo-stimulus has to be kept in mind in the low-demand conditions and compared to a single go stimulus (see Section Go/nogo task). (c) The N2 component should be more negative for high vs. low aversive reinforcement conditions. (d) Higher total reasoning scores as well as Gf and verbal reasoning ability were expected to intensify conflict monitoring resulting in a more negative N2 component. (e) With regard to personality, we expected that higher Trait-BIS individuals show a more intense conflict monitoring (i.e., more negative N2 component) under high vs. low aversive reinforcement.

## Method

### Participants

A total of *N* = 97 right-handed students of the University of Hamburg, Germany, took part in this study. The sample of the present study is completely independent of the sample reported in Leue et al. (2012a). Due to a large number of artifacts in the EEG data (for artifact rejection criterion see below), *n* = 7 participants had to be excluded because they had less than 25 artifact-free nogo epochs in each of the four task conditions (see below). Thus, *N* = 90 participants (46 male) were available for statistical analysis (age: *M* = 26.63 years, *SD* = 4.05; range: 18–42 years). All participants took part voluntarily in this study and gave written informed consent at the beginning of the study. The ethical board of the German Foundation of Psychologists evaluated the experimental protocol of the present study among a larger set of project studies using go/nogo tasks. No ethical concerns of the experimental protocols have been raised.

### Measures

Participants filled in the German version of the BIS/BAS scales (Strobel et al., 2001), which was originally published by Carver and White (1994). The BIS/BAS scales comprise 7 trait-BIS items and 13 trait-BAS items. All items can be answered on a 4-point Likert-type scale. The trait-BIS scale measures individual differences of aversiveness sensitivity and the trait-BAS scale measures individual differences of reward sensitivity. Cronbach’s alpha coefficients of both subscales were moderate (trait-BIS: 0.81, total trait-BAS: 0.74,) and widely comparable to prior studies (Carver and White, 1994). In order to give a more complete account of rRST and because problems with the suppression of predominant responses have been shown for high trait-BAS individuals (Lange et al., 2012) it was also explored whether individual differences in the sensitivity for appetitive reinforcement (trait-BAS) were related to conflict monitoring although no specific assumptions on this relation are investigated here (but see Gray and Braver, 2002; Leue et al., 2012b). The trait-BIS scale and the trait-BAS scale did not significantly correlate, *r*(90) = 0.04, *p* = 0.70 (two-tailed).

Total reasoning ability was assessed with a verbal analogy task (items 21–40, Cronbach’s alpha: 0.59), a numerical calculation task (items 61–80; Cronbach’s alpha: 0.81), and a figural matrices task (items 161–180; Cronbach’s alpha: 0.65) of the basic module of the Intelligence-Structure-Test 2000 R (I-S-T 2000 R; Liepmann et al., 2007; Beauducel et al., 2010). Since the figural matrices correspond exactly to other tests like Raven’s Advanced Progressive Matrices (Raven et al., 1998) that are widely used as measures of Gf (e.g., Burgess and Braver, 2010), the figural matrices are described as Gf measures in the following. Cronbach’s alpha of the total reasoning scores (0.82) was moderate. Handedness was investigated with the Edinburgh Handedness Inventory (Oldfield, 1971). All participants included in this study reported to be right-handed.

### Go/nogo task

**Figure 2:**
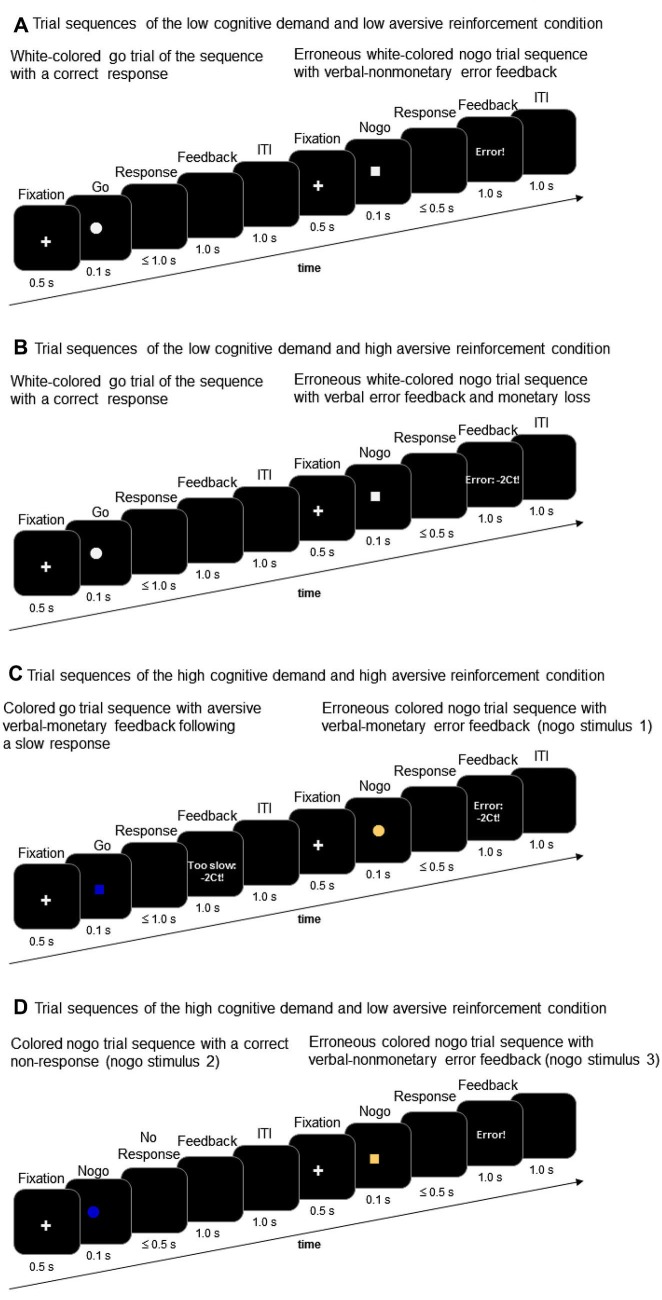
**Trial sequences of go and nogo trials in the low working-memory-related cognitive demand condition (A and B) and in the high working-memory-related cognitive demand condition (C and D) along with examples of low aversive reinforcement (i.e., verbal error feedback) and high aversive reinforcement (i.e., verbal error feedback and monetary loss)**s.

With regard to trial sequence and trial timing, the go/nogo task of this study corresponds to the task description presented in prior studies (Amodio et al., 2008; Leue et al., 2012a). Go and nogo stimuli were presented in an 80:20 ratio with 200 go stimuli and 50 nogo stimuli comprising each task condition. Go and nogo stimuli were geometric forms, that is either a square or a circle. The present go/nogo task combined a low vs. high cognitive demand condition and a low vs. high aversive reinforcement condition in a within-subjects-design. Each task block incorporated the combination of a demand level and a reinforcement level resulting in the following task conditions: (a) low demand/low aversive reinforcement; (b) low demand/high aversive reinforcement; (c) high demand/low aversive reinforcement; (d) high demand/high aversive reinforcement. Cognitive demand was manipulated by means of working memory load. The low demand condition required participants to respond to a white colored geometric shape (go stimulus, e.g., a white square) and to withhold responses to the other white colored geometric shape (nogo stimulus, e.g., white circle, Figures 2A and B). The high demand condition required participants to respond to one pre-defined colored go stimulus (e.g., blue colored square) and to withhold responses to three different nogo stimuli that varied in color and shape across task conditions (e.g., nogo stimuli could be a blue circle, a brown square, or a brown circle, Figures 2C and D).

Whereas the low cognitive demand condition required less working memory load because participants had to keep in mind only the shape of the pre-defined nogo stimulus, the high demand condition required more intense memory load because both color and shape must be inspected in order to respond correctly. Low vs. high levels of aversive reinforcement were realized in accordance with our prior studies on conflict monitoring in that low aversive reinforcement was defined as a negative verbal feedback such as “too slow” or “error” (Leue et al., 2012a,b). Too slow feedback was presented when responses to go stimuli occurred between 500 and 1000 ms post-stimulus. High aversive reinforcement was defined as a combination of aversive verbal and monetary reinforcement (for short: monetary loss) such as “too slow: −2 Ct” or “error: −2 Ct” (Leue et al., 2012a,b). A trial sequence comprised a 1000 ms lasting fixation cross followed by a 100 ms lasting go or nogo stimulus. Responses were required within 500 ms after stimulus-offset although responses to go stimuli were recorded up to 1000 ms stimulus-offset in order to determine “too slow” responses. In case of erroneous responses to nogo stimuli, non-responses to go stimuli or in case of too slow responses to go stimuli negative feedback (low vs. high depending on task condition, Figure 1) was provided for 1000 ms. In case of correct responses within the 500 ms interval to go stimuli and in case of correct non-responses to nogo stimuli, respectively, no feedback was provided and the screen remained black for 1000 ms. The inter-trial-interval was 1000 ms.

### Procedure

Participants were recruited through announcements on a bulletin board and an electronic platform announcing recent research projects at the University of Hamburg, Germany. Participants who were interested in this study were instructed in a telephone call to omit alcohol use, to avoid unusual caffeine and nicotine consumption, and to avoid taking any medication the day before EEG recording. All participants reported that they have never had any neurological disorder. At the beginning all participants gave written informed consent and were then seated in a comfortable chair. The chair was placed about 95 cm from the 20 inch flat screen that was used to present the go/nogo task. The room where the EEG was recorded was electrically shielded, sound-attenuated, and well-lit. Presentation V12.1 (Neurobehavioral Systems, Albany, NY) was used to present the go/nogo task. All participants performed four practice trials in the low demand conditions (i.e., watching the go and the nogo stimulus twice) and eight practice trials in the high-demand condition. Participants were informed in the instruction that erroneous responses to nogo stimuli or too slow responses to go stimuli (i.e., responses occurring between 500 and 1000 ms after stimulus-offset) would result in verbal error feedback (“error!”) or too slow feedback (“too slow!”) in the low aversive reinforcement conditions. Aversive verbal feedback and monetary loss would occur in case of erroneous responses (“error: −2 Ct!”) or too slow responses (”too slow: −2Ct!”) in the high aversive reinforcement conditions (Ct = cent). Participants were also informed that no feedback would be given for correct responses to go stimuli and correct non-responses to nogo stimuli. Doubling the number of practice trials in the high demand conditions was due to the fact that participants should have the opportunity to watch the go trial and the three nogo trials twice —as in the low demand condition. The experimenter sat in an adjacent room, where EEG data were saved to disk. The experimental session lasted approximately 100 min and was divided into four 15 min blocks during which EEG was recorded. Each of the four task blocks comprised one of the four task conditions of the go/nogo experiment. After finishing the task, participants received a basic payment of 15 € and an additional payment of maximal 10 € depending on their performance during the go/nogo task conditions with monetary loss, resulting in a maximum payment of 25 €. The order of the four task conditions was counter-balanced so that each of the four task conditions was once presented in the first, second, third, and fourth position, respectively. Among these four task conditions go and nogo shapes were alternated so that participants were asked to respond to a circle in one task condition (go stimulus), whereas a square was the go stimulus in the next condition. Participants were randomly assigned to one of the four task condition sequences (sequence 1: *n* = 22, sequence 2: *n* = 22, sequence 3: *n* = 23, sequence 4: *n* = 23).

### EEG recording and processing

**Figure 3:**
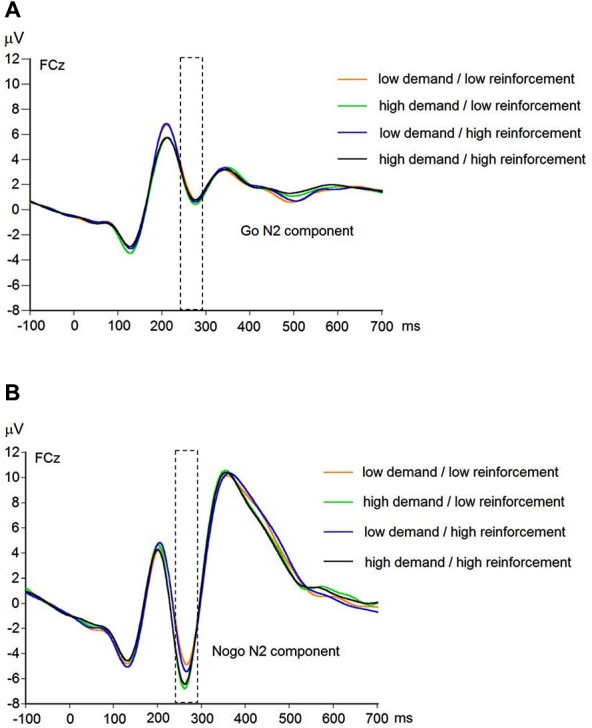
**Raw waveforms of go trials (A) and nogo trials (B) for combined working-memory-related cognitive demand and aversive reinforcement levels at FCz (*N* = 90)**.

**Figure 4:**
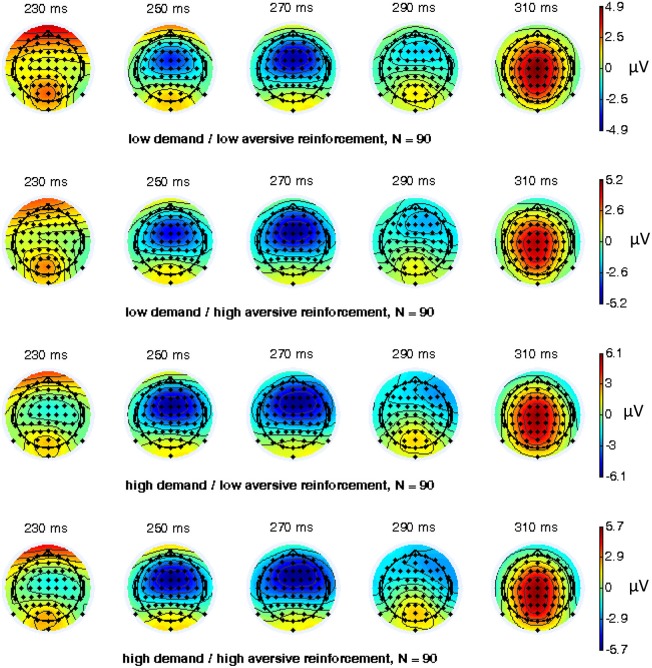
**Topographical maps for combined working-memory-related cognitive demand and aversive reinforcement levels of 64 electrode sites of the nogo N2 component (*N* = 90)**.

EEG recording, quantification, and analysis were conducted with reference to the guidelines for the study of human ERPs (Picton et al., 2000). EEG was recorded with 64 scalp active electrodes from the ActiveTwo BioSemi system (BioSemi, Amsterdam, Netherlands) based on the extended 10/20 system (Chatrian et al., 1988). The electrooculogram (EOG) was recorded from two horizontal electrodes placed beyond the epicanthi of both eyes and one vertical electrode located approximately 1 cm below the right eye. As per BioSemi’s design, the ground electrode during acquisition was formed by the Common Mode Sense active electrode and the Driven Right Leg passive electrode. All bioelectric signals were digitized on a laboratory computer using ActiView software (BioSemi). The impedances were below 25 kΩ during EEG recording. The EEG was sampled at 512 Hz. Off-line analysis was performed by using EEGLab v11.4.0.3b based on MATLAB 7.14.0.739 (The MathWorks, 2012). All data were band-pass filtered (1–15 Hz, Leue et al., 2013) and were re-referenced to averaged mastoids. Independent Component Analysis (ICA; an automated infomax decomposition) was applied to correct for ocular artifacts. Further technical and muscle artifacts were rejected when the EEG signal exceeded ±75 μV. Artifact-free epochs were separately segmented for go and nogo trials with correct responses lasting 700 ms after stimulus onset with a pre-stimulus baseline of 100 ms. The percentage of ERP epochs with artifacts varied between 5.6% and 13.8% indicating a good EEG signal quality. Comparable to prior studies, the N2 component peaked about 270 ms post-stimulus in an interval between 250–290 ms post-stimulus (Figure 3) and has a clear fronto-central topography (Figure 4). The N2 amplitude was quantified as the mean amplitude in the interval between 250–290 ms post-stimulus. The mean N2 amplitude was analyzed in this study because it is more reliable than the peak N2 amplitude (i.e., peak minus baseline) in go/nogo tasks with about 20–30 nogo epochs per task condition (Luck, 2005; Leue et al., 2013).

### Statistical analysis

Statistical analysis was performed using SPSS 22.0. A repeated measures ANCOVA was conducted for the behavioral data (go response times and sensitivity d′). Sensitivity d′ was computed according to Stanislaw and Todorov (1999). Sensitivity d′ has been introduced in the signal detection theory as a parameter of response accuracy taking the relative frequency of correct responses and commission errors into account. Conflict monitoring should result in the recruitment of top-down control and successful behavioral adaptation (i.e., more correct responses and fewer incorrect responses). The repeated measures ANCOVAs of the behavioral data included Go-Nogo stimulus type (2 levels: Go vs. Nogo), Cognitive demand (2 levels: low vs. high), and Aversive reinforcement (2 levels: low vs. high) as repeated measures factors. Gender and Task condition sequence were used as between-subjects factors in the repeated measures ANCOVA. Mean-centered trait-BIS, trait-BAS, and total reasoning sum scores were entered as continuous variables (MacCallum et al., 2002). In addition, we conducted the same repeated measures ANCOVA including the three reasoning subscales instead of the total reasoning sum scale.

The repeated measures ANCOVA of the mean nogo N2 amplitude included Region (3 levels of mean N2: frontal (F3, Fz, F4), central (C3, Cz, C4), parietal (P3, Pz, P4) and Laterality (3 levels of mean N2: left (F3, C3, P3), middle (Fz, Cz, Pz), and right (F4, C4, P4))) as repeated measures factors as well as Go-Nogo stimulus type, Cognitve demand, and Aversive reinforcement. Gender and task condition sequence were again the between-subjects factors. Mean-centered trait-BIS, trait-BAS, and total reasoning sum scores were entered as continuous variables (MacCallum et al., 2002). Again, we conducted the same repeated measures ANCOVA including the three reasoning subscales instead of the total reasoning sum scale. In all repeated measures ANCOVAs Greenhouse-Geisser Epsilon has been used in order to correct for sphericity violation of the repeated measures factors. Partial eta square (*η_p_^2^*) has been reported to indicate the effect size of main effects and interactions.

## Results

### Results of errors

A non-parametric test on the number of error of omissions and error of commissions (collapsed across the four task conditions) indicated that the participants made more commission errors on nogo trials (*M* = 4.40, *SD* = 3.15) than omission errors on go trials (*M* = 3.95, *SD* = 2.95), Wilcoxon-test, *p* < 0.05. As the descriptive statistics indicate, commission errors and omission errors were rather seldom.

### Results of go response times

**Figure 5:**
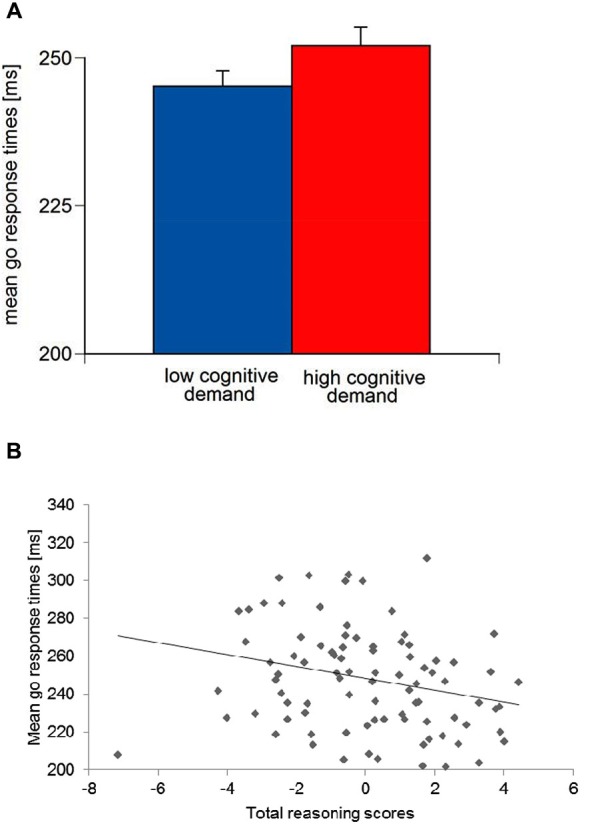
(**A**) Means and standard errors of mean go response times for low vs. high working-memory-related cognitive demand and (**B**) scatter plot for mean go response times and total reasoning scores.

For mean go response times, an Aversive reinforcement main effect was observed, *F*_(1,79)_ = 4.28, *p* < 0.05, *η_p_^2^* = 0.05. A significant Cognitive demand main effect, *F*_(1,79)_ = 15.48, *p* < 0.01, *η_p_^2^* = 0.16, indicated longer go response times in high Cognitive demand compared to low Cognitive demand conditions (Figure 5A). Go response times in the monetary loss feedback conditions were longer (*M* = 250.42 ms, *SE* = 2.95) compared to the verbal error feedback conditions (*M* = 246.80 ms, *SE* = 2.83).

With regard to the main effect of go response times was significant for the total Reasoning score, *F*_(1,79)_ = 7.42, *p* < 0.01, *η_p_^2^* = 0.09. ANCOVA parameter estimates suggested that higher total Reasoning ability were associated with shorter go response times (Figure 5B). Moreover, an Aversive reinforcement × Trait-BIS interaction was observed, *F*_(1,79)_ = 3.85, *p* < 0.05, *η_p_^2^* = 0.05, but no Trait-BIS main effect on go response times, *F*_(1,79)_ = 2.02, *p* = 0.16. Parameter estimates for Trait-BIS revealed that individuals with higher Trait-BIS scores had longer go response times with increasing Aversive reinforcement conditions. Moreover, an Aversive reinforcement × Trait-BIS × Gf interaction for go response times, *F*_(1,71)_ = 3.99, *p* = 0.05, *η_p_^2^* = 0.05, was observed. ANCOVA parameter estimates indicated that individuals with higher Trait-BIS and higher Gf scores had shorter go response times with increasing Aversive reinforcement (i.e., difference scores of high Aversive reinforcement minus low Aversive reinforcement have been established).

### Results of sensitivity d′

With regard to sensitivity d′, we observed an Aversive reinforcement main effect, *F*_(1, 79)_ = 7.72, *p* < 0.01, *η_p_^2^* = 0.09, with a higher sensitivity d′ occurring for high Aversive reinforcement (*M* = 4.29, *SE* = 0.08) vs. low Aversive reinforcement (*M* = 4.15, *SE* = 0.08). The Cognitive demand main effect for sensitivity d′ was not significant, *F*_(1,79)_ = 0.01, *p* = 0.99. The tendency of a Cognitive demand × Aversive reinforcement interaction, *F*_(1,79)_ = 3.48, *p* = 0.07, *η_p_^2^* = 0.04, suggested a significant Aversive reinforcement main effect under low Cognitive demand indicating an increase of sensitivity d′ from low Aversive reinforcement (*M* = 4.10, *SE* = 0.09) to high Aversive reinforcement (*M* = 4.34, *SE* = 0.09), *F*_(1,79)_ = 8.06, *p* < 0.01, *η_p_^2^* = 0.09. A Trait-BAS main effect for sensitivity d′, *F*_(1,79)_ = 5.40, *p* < 0.05, *η_p_^2^* = 0.06, indicated a higher sensitivity d′ with higher Trait-BAS scores. For sensitivity d′, we did not observe any main effects or interactions with Trait-BIS, total Reasoning or the Reasoning subscales.

### Results of the N2 amplitude

**Figure 6:**
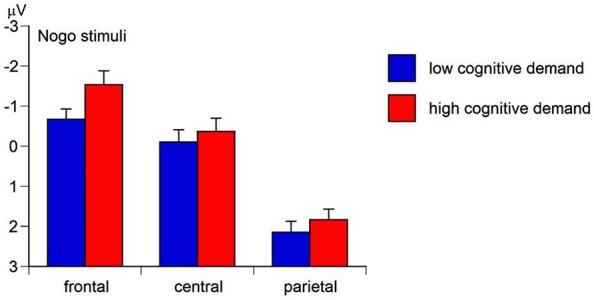
**Means and standard errors for the region x working-memory-related cognitive demand interaction of the mean nogo N2 amplitude (negative values represent more intense conflict monitoring)**.

The Region main effect of the mean N2 amplitude was significant, *F*_(2,158)_ = 129.49, *p* < 0.01, ɛ = 0.62, *η_p_^2^* = 0.62. Simple contrasts revealed the most pronounced mean N2 amplitude at frontal sites (*M*= −0.97 μV, *SE* = 0.28), *F*_(1,79)_ = 141.34, *p* < 0.01, *η_p_^2^* = 0.64, and central sites (*M*= 0.14 μV, *SE* = 0.25), *F*_(1,79)_ = 153.66, *p* < 0.01, *η_p_^2^* = 0.66, compared to parietal sites (*M*= 2.13 μV, *SE* = 0.21). The Go-Nogo stimulus main effect was significant, *F*_(1,79)_ = 281.20, *p* < 0.01, *η_p_^2^* = 0.78, with the Go N2-amplitude (*M* = 2.68 μV, *SE* = 0.24) being less pronounced than the Nogo N2-amplitude (*M* = −1.81 μV, *SE* = 0.28) suggesting a more intense conflict monitoring to nogo stimuli than to go stimuli (hypothesis a). As predicted in hypothesis b, the Cognitive demand main effect was significant, *F*_(1,79)_ = 17.85, *p* < 0.01, *η_p_^2^* = 0.18, indicating a less positive N2 amplitude (i.e., more intense conflict monitoring) in high-demand conditions (*M*= 0.78 μV, *SE* = 0.20) compared to low-demand conditions (*M*= 0.10 μV, *SE* = 0.27). Moreover, the Region × Go-Nogo-Stimulus × Cognitive demand interaction of the N2 amplitude was significant, *F*_(2,158)_ = 8.02, *p* < 0.01, ɛ = 0.61, *η_p_^2^* = 0.09. Separate ANCOVAs for Go and Nogo stimuli revealed a significant Region × Cognitive demand interaction for Nogo stimuli, *F*_(2,158)_ = 12.96, *p* < 0.01, ɛ = 0.66, *η_p_^2^* = 0.14, but not for Go stimuli, *F* = 1.01, ns. For Nogo stimuli, the N2 amplitude was most negative at fronto-central sites in high Cognitive demand conditions compared to low Cognitive demand conditions (Figure 6). This suggests that higher cognitive demand served as an aversive teaching signal during ACC-related conflict monitoring (Botvinick, 2007).

The Aversive reinforcement main effect of the N2 amplitude was not significant (hypothesis c), *F*_(1,79)_ = 0.41, *p* = 0.53. Neither the interaction of Aversive reinforcement × Cognitive demand, *F*_(1,79)_ = 1.23, *p* = 0.27, the Region × Aversive reinforcement interaction, *F*_(1,79)_ = 0.01, *p* = 0.97, nor the Region × Aversive reinforcement × Cognitive demand interaction, *F*_(1,79)_ = 0.45, *p* = 0.55, was significant.

**Figure 7:**
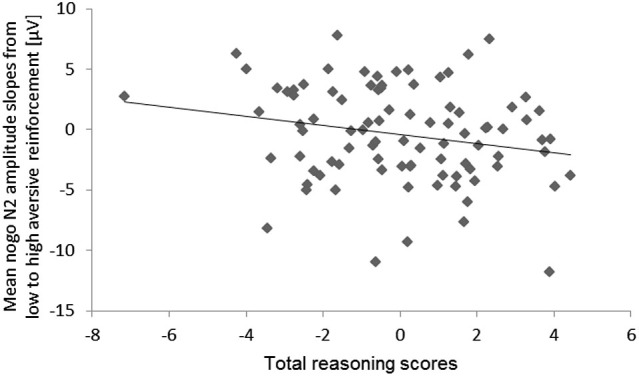
**Scatter plot for the total reasoning scores and the mean N2 amplitude slopes from low to high aversive reinforcement (collapsed across electrode sites)**.

With regard to individual differences, we observed a tendency of an Aversive reinforcement × total Reasoning interaction (hypothesis d), *F*_(1,79)_ = 3.74, *p* = 0.06, *η_p_^2^* = 0.05. This interaction indicated that higher total Reasoning scores were associated with a more pronounced decrease of the mean N2 amplitude (more negativity) with increasing Aversive reinforcement. For illustration of this N2 effect a scatter plot of the high minus low Aversive reinforcement difference was computed (Figure 7).

In order to investigate the specific effects of Gf and Verbal reasoning on conflict monitoring (hypothesis d) repeated measures ANCOVA including the Reasoning subscales and allowing for interactions between covariates were performed. A significant main effect was observed for Verbal reasoning, *F*_(1,71)_ = 4.99, *p* < 0.05, *η_p_^2^* = 0.07. Parameter estimates unexpectedly revealed that higher Verbal reasoning scores were related to more positive mean N2 amplitudes. The Go-Nogo × Gf interaction, *F*_(2, 158)_ = 9.42, *p* < 0.01, *η_p_^2^* = 0.12, could be traced back to a Gf main effect for nogo stimuli, *F*_(1,71)_ = 7.45, *p* < 0.01, *η_p_^2^* = 0.10, but not for go stimuli, *F* < 1, ns. Parameter estimates for Gf revealed that higher Gf scores were related to more negative nogo N2 amplitudes. An interaction of Gf × Verbal scores for the N2 amplitude was not observed. However, a significant main effect for Verbal reasoning occurred, *F*_(1,71)_ = 4.99, *p* < 0.05, *η_p_^2^* = 0.07. Parameter estimates unexpectedly revealed that higher Verbal reasoning scores were related to more positive mean N2 amplitudes. Finally, a Go-nogo × Aversive reinforcement × Trait-BIS interaction was significant (hypothesis e), *F*_(1,71)_ = 7.90, *p* < 0.01, *η_p_^2^* = 0.10. Again separate ANCOVAs for Go and Nogo stimuli were conducted and could be traced back to a significant Aversive reinforcement × Trait-BIS interaction of the nogo N2 amplitude, *F*_(1,71)_ = 3.98, *p* = 0.05, *η_p_^2^* = 0.05, but not of the go N2 amplitude, *F*_(1,71)_ = 0.07, *p* = 0.80. Parameter estimates for Trait-BIS suggested that higher Trait-BIS scores were associated with more negative nogo N2 amplitudes in conditions with low aversive reinforcement.

## Discussion

The present study investigated variations of the conflict monitoring intensity by means of working-memory-related cognitive demand (low vs. high) and aversive reinforcement (low vs. high) in a go/nogo task. The dorsal ACC is a prominent area in the brain that monitors effects of task difficulty and aversive reinforcement during conflict monitoring (Gray and McNaughton, 2000; Botvinick et al., 2004; Ridderinkhof et al., 2004). Moreover, the frontal N2 component as a reliable indicator of ACC-related conflict monitoring intensity (Amodio et al., 2008; Leue et al., 2013) was thought to indicate variations of conflict monitoring. In accordance with our prediction (a) the N2 amplitude was more negative following nogo compared to go stimuli. With regard to cognitive demand, we observed—as expected—(b) a more negative N2 amplitude for higher vs. lower cognitive demand in nogo trials, but not in go trials. (c) The N2 amplitudes did not differ in conditions with high vs. low aversive reinforcement and we did not observe interactions of aversive reinforcement × cognitive demand. (d) Higher reasoning scores were related to more negative nogo N2 amplitudes (i.e., more intense conflict monitoring) with increasing aversive reinforcement. Moreover, we observed that higher Gf scores were associated with more intense conflict monitoring especially to nogo trials, whereas higher verbal reasoning scores were related to less intense conflict monitoring. Although no individual differences of trait-BIS alone were observed for the nogo N2 amplitude, (e) more pronounced conflict monitoring was found in individuals with higher trait-BIS and verbal reasoning scores and in higher trait-BIS individuals in low aversive reinforcement conditions. Moreover, higher trait-BIS individuals showed a slight response times slowing with increasing aversive reinforcement. In order to elucidate variations of the conflict monitoring intensity and subsequent processes like reactive control, adjustment flexibility, and processing efficiency we subsequently discuss these N2 findings in association with the behavioral findings.

### Working-memory-related cognitive demand and conflict monitoring

First of all, although fMRI data show a pronounced activation of the dorsal ACC indicating intense conflict monitoring when cognitive demand is high (e.g., Vogt, 2009; Shenhav et al., 2013; Hernandez Lallement et al., 2014), the modulation of the fronto-central nogo N2 amplitude of the ERP by means of working-memory-related cognitive demand is a new finding. Our nogo N2 findings support Botvinick’s (2007) prediction that higher cognitive demand intensifies conflict monitoring. Moreover, go response times suggest that experimentally manipulated high cognitive demand induced a costly process because response times to go stimuli were longer in high vs. low demand conditions. However, response slowing was only so intense that participants did not evoke too many omission errors or too slow responses (i.e., response times > 500 ms), which would have resulted in “too slow” feedbacks and depending on task condition also in monetary loss. Conclusively, participants demonstrated a behavioral adjustment to go stimuli in conjunction with an intensified conflict monitoring under high cognitive demand (Weldon et al., 2013). We conclude that individuals have possibly less cognitive control under higher working-memory-related demand but show an intensified conflict monitoring in conjunction with response times slowing to prevent errors.

In contrast to Leue et al. (2012a), we did not observe the most negative N2 amplitudes in conditions with higher aversive reinforcement and lower demand. In the present data, working-memory-related demand overruled effects of aversive reinforcement on conflict monitoring intensity, because—again in contrast to Leue et al. (2012a)— we did not find a Region × Aversive reinforcement interaction indicating more negative N2 amplitudes in the aversive reinforcement condition at frontal sites. Thus, we conclude that working-memory-related demand is a more intense manipulation of cognitive demand than the go-nogo ratio that was applied as a manipulation of cognitive demand in other studies (Schacht et al., 2009; Leue et al., 2012a). The stronger effect of higher working memory load on the conflict monitoring intensity suggests that more intense cognitive demand detracts cognitive capacity from aversive signal processing.

### Reasoning ability and the flexibility to engage in conflict monitoring

The second main result of this study refers to the role of aversive reinforcement during conflict monitoring and its association with individual differences of total reasoning scores. Individuals with higher total reasoning ability demonstrated a more intense conflict monitoring with increasing aversive reinforcement and across task conditions they responded faster. These findings suggest, in accordance with Weldon et al. (2013), that individuals with higher total reasoning scores demonstrated a more flexible engagement to conflict monitoring because they increased their conflict monitoring when necessary and were faster than individuals with lower total reasoning scores. Moreover, the effect that individuals with higher reasoning ability intensified their conflict monitoring with increasing aversive reinforcement (i.e., more negative N2 amplitude) might suggest that the intensified conflict monitoring in individuals with higher reasoning ability results from a less intense recruitment of cognitive control. Thus, conflict monitoring intensity and cognitive control are reciprocally linked (cf. Botvinick et al., 1999; Kerns et al., 2004). That is, when conflict monitoring intensity is high cognitive control is low. Although conflict adaptation (Botvinick et al., 2001; Ullsperger et al., 2005; Clayson and Larson, 2012) and the reciprocal association of conflict monitoring and recruitment of cognitive control (Botvinick et al., 1999; Kerns et al., 2004) have not been investigated in this study, we preliminarily suggest based on the N2 data and the behavioral data that the association of conflict monitoring and behavioral adjustments as observed by means of the behavioral data might indicate reactive control (Braver et al., 2007; Braver, 2012). In the dual-mechanism of control, reactive control has been related to transient activation of the PFC or related brain systems like ACC (Braver et al., 2007). Reactive control is activated specifically in the moment an intention arises (“just-in-time” form of interference resolution) and it is reactivated by a trigger (e.g., aversive feedback) at another time (Braver et al., 2007). Thus, our data suggest that individuals with higher total reasoning scores had an enhanced conflict monitoring (N2 amplitude) especially when aversive reinforcement to errors was high (i.e., monetary loss) and they demonstrated more intense behavioral adjustments like fasting response times. Although we could not demonstrate the reciprocal relationship between conflict monitoring intensity and recruitment of cognitive control explicitly here, we could observe the subsequent behavioral consequences of an enhanced conflict monitoring. This let us interpret the conjunction of N2 data and behavioral data in individuals with higher reasoning scores as some evidence of reactive control. By suggesting this link between conflict monitoring and behavioral adjustment we do not implicitly equate conflict monitoring and cognitive control.

Similarly, individuals with combined higher verbal reasoning scores and higher trait-BIS scores showed a more intense conflict monitoring especially to nogo stimuli. However, this intensification of conflict monitoring (i.e., more negative nogo N2 amplitude) co-occurred with a slight response times slowing in higher trait-BIS individuals and, thus, was associated with a more cautious response tendency in higher trait-BIS individuals. It could be that the intensified conflict monitoring of participants with higher trait-BIS and higher verbal reasoning scores reflects an intensified processing of aversive reinforcement which is possibly induced by more pronounced rehearsal of the verbal self-instruction to minimize monetary loss. The enhanced conflict monitoring of higher trait-BIS individuals with higher verbal reasoning scores may indicate that especially the combination of aversive feedback avoidance in higher trait-BIS individuals (Gray and McNaughton, 2000; Corr, 2008; Leue and Beauducel, 2008) and the probably more intense rehearsal of verbal self-instructions results in an intensified conflict monitoring. In summary, conflict monitoring was enhanced in individuals with higher verbal reasoning ability only when high aversive reinforcement co-occurs with higher sensitivity to aversive reinforcement (trait-BIS). This result illustrates the complexity of the relationship between personality and intelligence as it has already been discussed elsewhere (Demetriou et al., 2003).

Interestingly, higher verbal reasoning scores alone were associated with less intense conflict monitoring. This result was unexpected and needs to be replicated before it can be taken into account in further research. A tentative interpretation of this result could be that higher verbal reasoning leads to more disengagement from the genuine stimulus evaluation of the figural go/nogo task because the figural task requirements did not match the performance expectations of individuals with higher verbal reasoning ability. However, the Gf main effect of the nogo N2 amplitude suggested that Gf as assessed by means of figural stimulus material was related to a more intense conflict monitoring in a go/nogo task that provided figural stimulus material (squares and circles, respectively). Accordingly, the fact that both the measure of Gf and the go/nogo task were based on figural material might have enhanced the relation between Gf and conflict monitoring in the present go/nogo task. To highlight the predicted relations on cognitive and non-cognitive individual differences, experimentally manipulated cognitive demand and aversive reinforcement on the conflict monitoring intensity and behavioral adjustments, we summarized these relations in Figure 7.

Together, these findings demonstrate that cognitive control enhances more flexible adjustment to demanding situations in individuals with higher reasoning ability (Braver et al., 2007; Neubauer and Fink, 2009; Weldon et al., 2013). In contrast, more intense cognitive control in individuals with higher trait-BIS and higher verbal reasoning may be primarily related to just-in-time reactive aversive feedback processing, which might help to enhance conflict monitoring in order to prevent aversive feedback. Verbal reasoning enhances the aversive feedback processing in higher trait-BIS individuals so that in a task with varying levels of cognitive demand the effects of trait-BIS and aversive reinforcement are compatible with the idea of a more intense reactive control strategy (Braver et al., 2007) and a more intense processing of aversive feedback as postulated in rRST (Gray and McNaughton, 2000; Corr, 2008; Leue and Beauducel, 2008; Weldon et al., 2013).

### Limitations and future directions

Since the present go/nogo task focused on figural stimulus material, the role of verbal abilities during conflict monitoring needs to be further explored in tasks providing more verbal stimulus material. This is of special relevance, because the result of lower conflict monitoring in individuals with higher verbal reasoning ability was unexpected and in contrast with the higher conflict monitoring found for individuals with higher reasoning ability under higher aversive reinforcement. Moreover, Eysenck et al. (2007) proposed that more anxious individuals are prone to processing inefficiency (i.e., more resources are recruited to perform well) and Moser et al. (2013) demonstrated that Error-related Negativity (ERN) is an index of this inefficiency. In the context of response-related error monitoring, higher anxious individuals rely on more error monitoring resources than lower anxious individuals to achieve a comparable performance (or even perform worse than lower anxious individuals). Basten et al. (2012) demonstrated lower processing efficiency in high anxious individuals in a working memory task. Since Basten et al. (2012) found lower processing efficiency in high anxious individuals when a task requiring WMC is performed, it could be expected that reduced processing efficiency of higher anxious individuals might occur in the context of conflict monitoring when a more substantial amount of working memory demand is incorporated into a go/nogo task. Our data demonstrate that higher trait-BIS individuals intensify their conflict monitoring even in conditions with low aversive reinforcement (i.e., verbal error or too slow feedback). This finding parallels to Leue et al. (2012b) who also found the most intense conflict monitoring of higher trait-BIS individuals in conditions with less intense aversive reinforcement. Irrespective of task conditions, higher trait-BIS individuals showed a higher sensitivity d′ compared to lower trait-BIS individuals. These findings support the idea that higher trait-BIS individuals are less flexible in adapting their conflict monitoring intensity depending on cognitive demand requirements and negative consequences. Instead they invest more resources in advance to perform well. Thus, processing inefficiency of higher trait-BIS individuals can be already observed during conflict monitoring, not only during response-related error monitoring. The interplay of stimulus-related conflict monitoring as investigated in this study and response-related error monitoring in terms of processing inefficiency in anxious individuals needs to be explored in future studies. Finally, Donkers and van Boxtel (2004) argue that the N2 amplitude indicates conflict monitoring because more negative N2 amplitudes were not only observed to nogo stimuli but also to rarely occurring go stimuli, which did not require response inhibition. Moreover, Bruin et al. (2001) observed that the N2 amplitude was not affected by response priming in a go/nogo task. That is why the authors concluded that the N2 amplitude is not associated with response inhibition. Instead Bruin et al. (2001) suggested the frontal P3 to be an indicator of response inhibition (see also, Mennes et al., 2008). As becomes apparent from these studies there is evidence that the N2 amplitude represents rather conflict monitoring than response inhibition in go/nogo tasks. However, as long as those studies that simultaneously analyze and interpret stimulus-locked N2- and P3-findings are rare, it cannot be completely excluded that the N2 amplitude might be also conceived as an indicator of response inhibition.

### Conclusions

Our data suggest that the manipulation of working memory load is an effective manipulation of cognitive demand and conflict monitoring intensity in a go/nogo task. Conflict monitoring was in particular intensified under high cognitive demand resulting in a more negative fronto-central mean nogo N2 amplitude. Thus, cognitive demand leads to an intensified stimulus evaluation during conflict monitoring resulting in behavioral adjustments. Individuals with higher total reasoning ability showed a more intense N2-related conflict monitoring with increasing aversive reinforcement but shorter go response times. These findings suggest that individuals with higher reasoning ability show more intense working-memory-related conflict monitoring and subsequently more flexible behavioral adjustments. Higher fluid intelligence (Gf) as measured by figural abilities appeared to facilitate conflict monitoring in our figural go/nogo task, whereas verbal abilities did not contribute to a more intense conflict monitoring. Based on this finding, we draw the preliminary conclusion that more intense conflict monitoring might occur when specific reasoning abilities and stimulus material of a task match. Conflict monitoring to nogo stimuli was also more intense in individuals with higher trait-BIS and verbal reasoning scores, but response times to go stimuli in higher trait-BIS individuals were slightly slower with increasing aversive reinforcement. These findings indicate that an intensification of conflict monitoring in higher trait-BIS individuals is related to situations with externally applied aversive reinforcement but not directly associated with cognitive demand requirements. Moreover, avoidance of error feedback is predominant in higher trait-BIS individuals irrespective of required working-memory-related cognitive demand.

## Conflict of interest statement

The authors declare that the research was conducted in the absence of any commercial or financial relationships that could be construed as a potential conflict of interest.
